# Serum, Urine, and Fecal Metabolome Alterations in the Gut Microbiota in Response to Lifestyle Interventions in Pediatric Obesity: A Non-Randomized Clinical Trial

**DOI:** 10.3390/nu15092184

**Published:** 2023-05-04

**Authors:** Yujin Lee, Joo-Youn Cho, Ky Young Cho

**Affiliations:** 1 Department of Clinical Pharmacology and Therapeutics, Chungbuk National University College of Medicine and Hospital, Cheongju-si 28644, Chungcheongbuk-do, Republic of Korea; lee_yj@cbnuhctc.com; 2 CBNUH Cheongju-Osong National Advanced Clinical Trial Center, 77, Osongsaengmyeong-ro, Osong-eup, Heungdeok-gu, Cheongju-si 28161, Chungcheongbuk-do, Republic of Korea; 3 Department of Clinical Pharmacology and Therapeutics, Seoul National University College of Medicine and Hospital, Seoul 03080, Republic of Korea; 4 Department of Biomedical Sciences, Seoul National University College of Medicine, Seoul 03080, Republic of Korea; 5 Department of Pediatrics, Hallym University Kangnam Sacred Heart Hospital, Seoul 07441, Republic of Korea

**Keywords:** pediatric obesity, metabolome, gastrointestinal microbiome, clinical trial, multiomics, metabolic networks and pathways

## Abstract

Pediatric obesity is associated with alterations in the gut microbiota and its metabolites. However, how they influence obesity and the effect of lifestyle interventions remains unknown.. In this non-randomized clinical trial, we analyzed metabolomes and microbial features to understand the associated metabolic pathways and the effect of lifestyle interventions on pediatric obesity. Anthropometric/biochemical data and fasting serum, urine, and fecal samples were collected at baseline and after an eight-week, weight-reduction lifestyle modification program. Post-intervention, children with obesity were classified into responder and non-responder groups based on changes in total body fat. At baseline, serum L-isoleucine and uric acid levels were significantly higher in children with obesity compared with those in normal-weight children and were positively correlated with obesogenic genera. Taurodeoxycholic and tauromuricholic α + β acid levels decreased significantly with obesity and were negatively correlated with obesogenic genera. Branched-chain amino acid and purine metabolisms were distinguished metabolic pathways in the obese group. Post-intervention, urinary myristic acid levels decreased significantly in the responder group, showing a significant positive correlation with *Bacteroides.* Fatty acid biosynthesis decreased significantly in the responder group. Thus, lifestyle intervention with weight loss is associated with changes in fatty acid biosynthesis, and myristic acid is a possible therapeutic target for pediatric obesity.

## 1. Introduction

Pediatric obesity is a pressing global health problem, and the incidence rate continues to increase. It is linked to an increased risk of type 2 diabetes, hypertension, dyslipidemia, and metabolic syndrome [1,2]. Diverse evidence suggests that obesity is associated with alterations in the gut microbiota and its metabolites [3,4,5]. In human gut microbial composition, the number of gut microbial genes and their richness have been reported, showing a difference between obese individuals and non-obese individuals. The obese individuals show a low microbial richness [6]. Comparisons of gut microbiota in obese mice and lean mice have uncovered that obesity is related to changes in the relative abundance of the two dominant Phylum, Bacteroidetes and Firmicutes, and especially, the abundance of Bacteroidetes is reduced, and the abundance of Firmicutes is increased [4,7,8,9]. Additionally, like the microbiome, specific metabolites are significantly changed in obesity [10]. Plasma glutamate concentration is inversely correlated with abundance of Bacteroides thetaiotaomicron and proportionally correlated with weight gain [11]. Increased amino acids and some fatty acids, such as palmitic acid and stearic acid, are found in individuals with obesity [12]. In addition, short-chain fatty acids, which are metabolites produced by the microbiome, are reported to be related to weight change. An acetate is increased in weight loss individuals and inversely correlated to fasting insulin [13].

However, most microbiome studies have focused on changes in microbial compositions and not specific microbiota; a few have focused on metabolites [14,15,16]. Moreover, cross-sectional and interventional obesity studies have mainly been performed in adults rather than children [17]. Children with obesity are more likely to become obese in adulthood, accompanied by lifelong physical and mental problems [18,19,20]. Thus, studying pediatric obesity is essential to identify therapeutic targets before obesity complications develop. An integrative study that combines the analysis of gut microbiota and metabolome could be valuable in unraveling the complex interactions between gut microbiota and metabolites in pediatric obesity.

We previously reported microbial alterations after a lifestyle intervention in children with obesity [21]. In this study, by integrating analyses of metabolomes with microbial features, we aimed to understand the metabolic pathways underlying pediatric obesity and the effect of intervention, which could provide guidance for the treatment of obesity.

## 2. Materials and Methods

### 2.1. Study Design and Population

This non-randomized clinical trial was conducted in accordance with the Declaration of Helsinki and approved by the Institutional Review Board of Hallym University Kangnam Sacred Heart Hospital (No. 2018–06–026-003). Written informed consent was obtained from all participants and parents (for all participants under the age of 18 years before the start of the study). This study followed the Transparent Reporting of Evaluations with Nonrandomized Designs (TREND) reporting guidelines [22].

We enrolled 50 children with obesity and 22 normal-weight children aged 7–18 years from 7 August 2018, to 21 August 2019, and followed them up from 1 October 2018, to 6 November 2019. Data were analyzed from June 2020 to November 2022. Obesity was defined as having a body mass index (BMI) ≥ 95th percentile from the age- and sex-specific Korean growth chart, whereas a BMI <85th percentile was defined as normal weight [23]. Participants who had underlying diseases such as congenital heart disease, chronic liver disease, and chronic inflammatory bowel disease were excluded. For participants with acute inflammatory diseases such as pneumonia, acute gastroenteritis, and influenza, the intervention was delayed by one month. The participants were asked not to take probiotics, antibiotics, or steroids for a month before the visit. Anthropometric measurements, biochemical blood marker measurements, and body composition analysis were performed during the first and third hospital visits [24,25]. Before and after an eight-week weight-reduction program, serum, urine, and fecal samples were collected for metabolomic and metagenomic analyses and stored at −80 °C.

Over eight weeks, participants with obesity underwent a weight-reduction program three times under the observation of research nurses, dietitians, exercise professionals, and pediatric clinicians at the hospital. In this program, every child with obesity received individualized and feasible lifestyle modifications designed by pediatric clinicians, dietitians, and exercise professionals. Research nurses monitored the participants to follow the recommendations every two weeks. A detailed method to produce individualized lifestyle interventions was applied according to previous protocols [21]. In this study, adiposity was defined as the percentage (%) of total body fat measured through body composition analysis using an InBody 770 analyzer (Biospace Co., Ltd., Seoul, Republic of Korea). A participant is classified as a responder if their total body fat (%) decreases after the intervention, and as a non-responder if it increases or remains unchanged.

### 2.2. Metagenomics

The gut microbiota was characterized by the 16S rRNA gene amplicon sequencing of the V3–4 regions using an Illumina MiSeq platform (Illumina, San Diego, CA, USA). All raw 16S rRNA gene sequencing data have been deposited in the NCBI Sequence Read Archive under the accession number SUB7459302 (BioProject PRJNA633584). Metagenomics analyses are described in detail in a previous article [21].

### 2.3. Sample Preparation for Untargeted Metabolomics

All samples were prepared using a protocol from previous studies with minor modifications [26,27]. Frozen serum, urine, and fecal samples were thawed on ice. Quality control (QC) samples, obtained by pooling equal volumes (50 μL) of the serum and urine samples, and 50 μL of the first extracted solution of the fecal samples, were used to validate the stability of analytical performance and perform data filtering. For serum and urine samples, 50 μL was extracted using 1 mL of the N_2_-degassed first extraction solution (3:3:2, acetonitrile:isopropanol:H_2_O). For fecal samples, 1 mL of the first extraction solution was added into the sample via a mass-to-solution volume ratio of 50 mg. Then, samples were mixed for 10 min and centrifuged for 10 min at 18,945 RCF and 4 °C. A 450-µL aliquot of the supernatant was dried using SpeedVac for 6 h at 45 °C and 5.1 vacuum pressure. Dried samples were re-extracted with 450 μL of the N_2_-degassed second extraction solution (1:1, acetonitrile: H_2_O), then re-dried for 8 h under the same conditions used for the first extraction step. Dried samples were derivatized with methoxyamine (20 mg/mL in pyridine) at 30 °C for 90 min, and subsequently trimethylsilylated with a mixture of fatty acid methyl ester, which was used for the retention time index, in N-methyl-N-(trimethylsilyl)-trifluoroacetamide at 70 °C for 45 min. Finally, 1-μL aliquots were split-injected into an Agilent 7890 series gas chromatography system (Agilent, Santa Clara, CA, USA) coupled to a time-of-flight mass spectrometer (LECO Corp., St. Joseph, MI, USA) for untargeted metabolomics.

### 2.4. Serum Bile Acids

Quantification was performed using the Biocrates^®^ Bile Acid Kit (Biocrates Life Science AG, Innsbruck, Austria) with SCIEX liquid chromatography–tandem mass spectrometry (AB Sciex API 4000™) according to the manufacturer’s instructions.

### 2.5. Metabolomic Data Analysis

Chroma TOF version 4.72 (LECO Corp.) was used for peak extraction, peak alignment, peak deconvolution, and peak identification. Data processing and multivariate analysis were performed using MetaboAnalyst 4.0. Detected metabolic features with missing values greater than 50% were removed, and the remaining missing values were replaced with a limit of detection of 1/5 of the minimum positive value of each variable. Metabolic features were filtered out according to a relative standard deviation of greater than 30% in the QC samples. Filtered metabolic features were normalized by sum, and Pareto scaling was applied for multivariate analysis. Metabolic markers were selected using a *t*-test with a *p*-value cutoff of less than 0.05. Statistical analyses were performed using GraphPad Prism 7 (GraphPad Software, Inc., San Diego, CA, USA).

### 2.6. Identification of Metabolic Markers

The Human Metabolome Database (HMDB) (https://hmdb.ca/) and three commercially available libraries (NIST, LECO-Fiehn Rtx5, and Wiley 9) were used. After matching the mass fragments of the markers with the libraries, authentic standards were analyzed to compare the mass fragments. Marker and standard retention times were compared by calculating the relative retention index [26].

### 2.7. Correlation Analysis

Spearman correlation analysis was performed after the normality test. Relative abundances of microbes in the microbiome, metabolic markers, and clinical parameters (anthropometric measurements and biochemical blood markers) were included in the analysis, which was performed using the Corrplot package in R software (version 4.1.1, http://www.R-project.org). The *p*-value cutoff was 0.05. A network diagram was generated using MetaMapp and Cytoscape (version 3.5, http://www.cytoscape.org/).

### 2.8. Chemicals

The fatty acid methyl ester mixture used for the relative retention time index and the authentic standards used for the identification of significant metabolic markers were purchased from Sigma-Aldrich (St. Louis, MO, USA). Extraction solvents used for sample preparation, such as isopropanol, acetonitrile, and water (HPLC grade), were obtained from J.T. Baker Chemical Co. (Phillipsburg, NJ, USA). Pyridine, methoxamine hydrochloride (MeOX), and N-methyl-N-(trimethylsilyl) trifluoroacetamide were used for derivatization and purchased from Sigma-Aldrich. The Biocrates^®^ Bile Acid Kit was purchased from Biocrates Life Science AG.

### 2.9. Statistical Analysis

Paired normally distributed data were analyzed using the paired *t*-test following the Shapiro–Wilk test, while paired non-normally distributed data were analyzed using Wilcoxon’s signed-rank test. The independent normally distributed data were analyzed using t-test, and the independent skewed continuous data were analyzed using Wilcoxon’s rank-sum test. Categorical variables were analyzed using the chi-square test and presented as frequencies and percentages. Statistical analysis was performed using MetaboAnalyst 4.0 [28] and GraphPad Prism 7 (GraphPad Software, Inc., San Diego, CA, USA). Spearman correlation analysis was performed after the normality test. Correlation analysis was performed on relative abundances in the microbiome, metabolic markers, and clinical parameters using the Corrplot package in R (v. 4.1.1). The *p*-value cutoff was 0.05. A network diagram was generated using MetaMapp [29] and Cytoscape (version 3.5) [30]. The cutoff for false discovery rate-adjusted *p*-values (FDR-adjusted *p*-value) and *p*-values (*p*) was 0.05.

## 3. Results

### 3.1. Study Population

**Figure 1:**
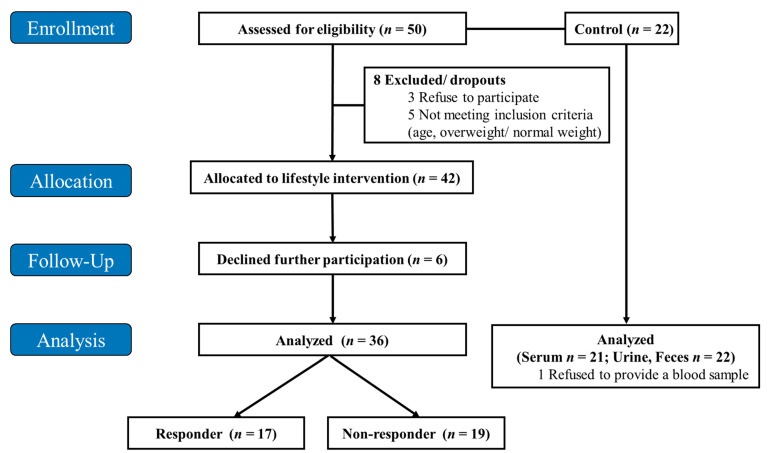
Flow diagram of the study design. The intervention was performed over eight weeks.

After the screening, 42 children with obesity participated in the first intervention; 36 of them completed the intervention, and 6 of them declined further participation. The 36 participants were classified into responder (*n* = 17) and non-responder (*n* = 19) groups according to changes in total body fat (%) after the intervention. In the children with obesity, fasting serum, urine, and fecal samples were collected from 17 responders and 19 non-responders. In 22 normal-weight children, only 21 serum samples were collected because one participant refused to provide a blood sample, but urine and fecal samples were collected from all 22 normal-weight children (Figure 1).

The mean age of all participants was approximately 10 years; 57–63% were male in each group (Table 1). BMI, total body fat (%), and glucose, alanine aminotransferase (ALT), and low-density lipoprotein (LDL) cholesterol levels were significantly higher in children with obesity (*p* < 0.05, Table 1). However, high-density lipoprotein (HDL) cholesterol levels showed the opposite trend (*p* < 0.05, Table 1). Before the intervention, the responder and non-responder groups did not show significant differences in anthropometric measurements and blood biochemical profiles. In the responder group, BMI, total body fat (%), and ALT decreased significantly after intervention (*p* < 0.05, Table 1). Conversely, weight, and BMI in the non-responder group increased significantly after the intervention, with no other significant changes observed in anthropometric/biochemical parameters (Table 1).

### 3.2. Baseline Metabolic Profiles in Children with Obesity

**Figure 2:**
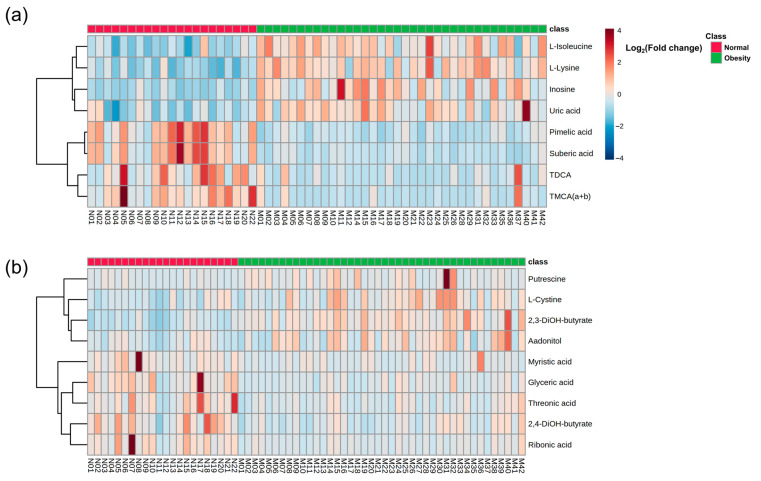
Heatmap showing (**a**) serum metabolites and (**b**) urinary and fecal metabolites that were significantly different between normal and obese groups. Significance was set at *p* < 0.05 *t*-test. Each colored cell represents a log_2_ fold change in metabolite levels compared with those in obesity/normal. TDCA, taurodeoxycholic acid; TMCA (a + b), tauromuricholic acid (α + β); 2,3-diOH-butyrate, 2,3-dihydroxybutanoic acid; 2,4-diOH-butyrate, 2,4-dihydroxybutanoic acid.

Metabolic profiles differed significantly between the normal and obese groups (*p* < 0.05 *t*-test, Figure 2 and Figure S1). Serum L-isoleucine, L-lysine, uric acid, and inosine levels were significantly higher in the obese group. In bile acid assays, taurodeoxycholic acid (TDCA) and tauromuricholic acid α + β (TMCA (α + β)), levels were significantly lower in the obese group (*p* < 0.05 *t*-test, Figure 2a and Table S1). Urinary L-cystine and 2,3-dihydroxybutanoic acid levels, as well as fecal myristic acid levels, were significantly higher in the obese group (*p* < 0.05 *t*-test, Figure 2b and Table S1).

### 3.3. Baseline Correlation Analyses

**Figure 3:**
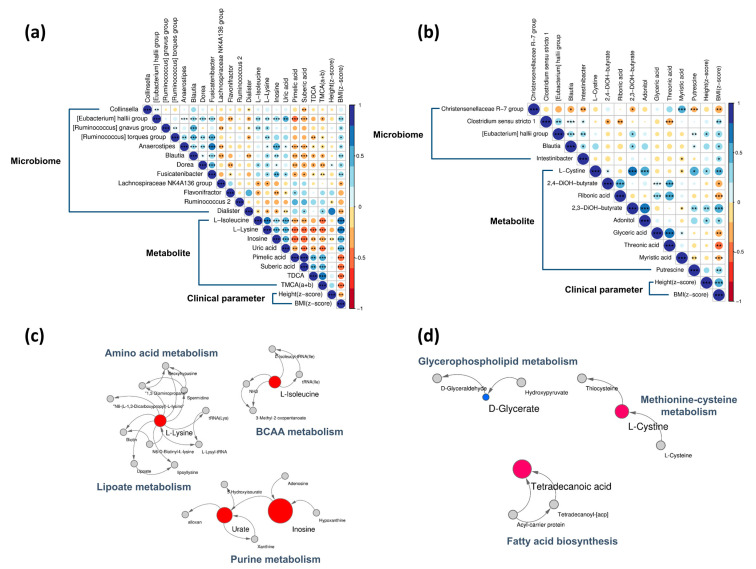
Baseline Spearman correlations and metabolic pathways. Correlations between the (**a**) serum normal and obese groups and (**b**) urine/feces normal and obese groups were determined. Pathways of (**c**) serum metabolites and (**d**) urinary/fecal metabolites that differed between normal and obese groups. In the Spearman correlations, circle size represents the correlation coefficient value. Blue indicates positive and red indicates negative correlations. Metabolic pathways are shown as nodes and lines. Nodes are colored based on significance with red for metabolites that increased with obesity, blue for metabolites that decreased with obesity, and gray for non-significant differences. Node sizes represent fold change (obese/normal). * *p* < 0.05, ** *p* < 0.01, *** *p* < 0.001.

In a previous study, we identified differences in microbial composition between normal and obese groups [21]. At the genus level, the relative abundances of the *Collinsella*, *Eubacterium hallii*, *Ruminococcus gnavus* groups, and *Dorea* were high, and those of *Flavonifractor*, *Ruminococcus 2*, and *Dialister* were low in the obese group [21]. Correlation analyses showed that the *Eubacterium hallii*, *Ruminococcus gnavus* groups, and *Dorea* as an obesogenic genus, were significantly and positively correlated with serum amino acids, inosine, uric acid, and BMI-Z score (BMIZ); however, they were negatively correlated with pimelic acid, TDCA, and TMCA (α + β) (*p* < 0.05, Figure 3a). The *Christensenellaceae R-7* group showed a significantly negative correlation with urinary 2,3-dihydroxybutyrate and fecal putrescine, but a significantly positive correlation with fecal myristic acid (*p* < 0.05, Figure 3b). Myristic acid levels were negatively correlated with BMIZ.

### 3.4. Baseline Metabolic Pathways

We found that amino acid metabolism, branched-chain amino acid (BCAA) metabolism, lipoate metabolism, and purine metabolism were prominent in the obese group at baseline (Figure 3c). Most metabolites of serum pathways increased in the obese group. Urinary and fecal metabolites, glycerophospholipid metabolism, methionine-cysteine metabolism, and fatty acid biosynthesis were increased in the obese group at baseline (Figure 3d).

### 3.5. Post-Intervention Metabolite Changes

**Figure 4:**
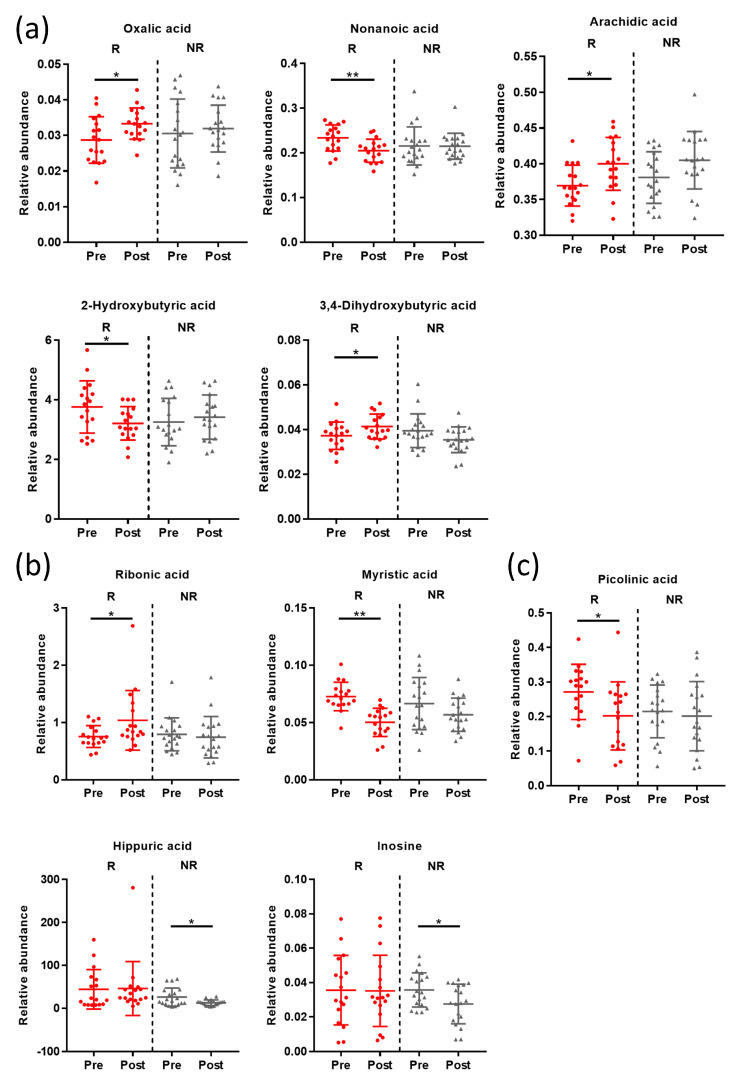
Whisker plots showing relative intensities of (**a**) serum, (**b**) urinary, and (**c**) fecal metabolites that were changed significantly by lifestyle interventions. Data are shown as means with standard deviations; each dot represents a sample. Pre, pre-intervention; Post, post-intervention; R, responder; NR, non-responder. * *p* < 0.05, ** *p* < 0.005 Wilcoxon’s signed-rank test.

Metabolic changes in the responder and non-responder groups differed significantly (*p* < 0.05 Wilcoxon’s signed-rank test; Figure 4 and Table S2). Oxalic acid, nonanoic acid, arachidic acid, 2-hydroxybutyric acid, and 3,4-hydroxybutyric acid in the serum samples were significantly changed in the responder group after intervention (*p* < 0.05 Wilcoxon’s signed-rank test, Figure 4a). In addition, levels of ribonic acid and myristic acid in the urinary samples were significantly changed after intervention in the responder group (*p* < 0.05 Wilcoxon’s signed-rank test, Figure 4b). However, the levels of hippuric acid and inosine decreased significantly after intervention in the non-responder group. Among fecal metabolites, picolinic acid decreased significantly in the responder group (*p* < 0.05 Wilcoxon’s signed-rank test, Figure 4c). Serum bile acid showed no significant changes between responder and non-responder groups after the intervention (data not shown). Unlike after the intervention, there was no significant difference in the metabolic profile between the responders and non-responders before the intervention.

### 3.6. Post-Intervention Correlation Analyses

In a previous study, we identified alterations in gut microbiota in response to lifestyle interventions [21]. In the responder group, at the phylum level, the relative abundance of Bacteroidetes decreased, whereas that of Firmicutes increased. At the genus level, *Bacteroides* decreased after intervention [21]. In the non-responder group, at the phylum level, Actinobacteria increased and Firmicutes decreased after the intervention; the trend was the opposite for the responder group [21].

**Figure 5:**
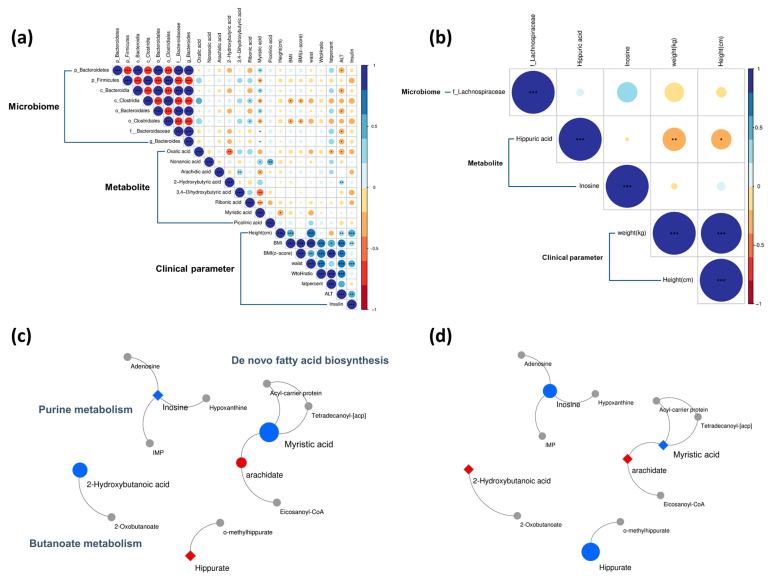
Post-intervention Spearman correlations and metabolic pathways. The Spearman correlations are shown for the (**a**) responder and (**b**) non-responder groups. In the correlations, circle size represents the magnitude of the correlation coefficient. Blue indicates positive correlations, and red indicates negative correlations. The metabolic pathways differed between (**c**) responder and (**d**) non-responder groups. Metabolites are indicated by color: red represents increased, blue represents decreased, and gray represents no change. Node size represents fold change. Circles are metabolites that significantly changed post-intervention and diamonds are metabolites that did not. * *p* < 0.05, ** *p* < 0.01, *** *p* < 0.001.

The altered microbiota, metabolites, and anthropometric/biochemical parameters were correlated with each other (Figure 5). *Bacteroides* showed a significant positive correlation with urinary myristic acid in the responder group. ALT was negatively correlated with *Bacteroides* and serum oxalic acid, but positively correlated with serum 2-hydroxybutyric acid. The phylum Firmicutes was negatively correlated with urinary myristic acid levels (*p* < 0.05, Figure 5a). In the non-responder group, urinary hippuric acid levels were negatively correlated with both weight and height (*p* < 0.05, Figure 5b).

### 3.7. Post-Intervention Metabolic Pathway Changes

Butanoate metabolism, de novo fatty acid biosynthesis, and purine metabolism were changed after the intervention (Figure 5). The level of 2-hydroxybutyric acid, which is involved in butanoate metabolism, was significantly lower in the responder group than in the non-responder group (Figure 5c). In de novo fatty acid biosynthesis, decreasing trends were similar in both groups; however, metabolites, including myristic acid and inosine, differed between the responder and non-responder groups, and the differences were statistically significant (Figure 5d). Furthermore, according to the functional metabolic analysis of gut microbiota by the Phylogenetic Investigation of Communities by Reconstruction of Unobserved States 2 in the previous study, the levels of the “Aspartate Superpathway” components were predicted to increase in the responder group after intervention [21]. Interestingly, in the global metabolite profiles in the current study, serum methionine, which is the intermediator of the aspartate superpathway, significantly decreased in the responder group after the intervention (Table S3).

## 4. Discussion

In this clinical trial designed to examine metabolic differences between normal-weight children and children with obesity, including between before and after lifestyle interventions, we found that imbalances in microbiota and metabolites were associated with both obesity and response to the intervention.

We found that serum isoleucine (a BCAA) was increased owing to obesity, which was consistent with previous studies showing increased isoleucine in obese rats and overweight/obese subjects [31,32], and this increase was positively correlated with anthropometric and biochemical parameters, such as BMI, total body fat (%), glucose, and triglycerides (TGs). Isoleucine levels have been shown to change with weight changes [33,34]. Increased isoleucine levels are predictive of insulin resistance and correlate with an increased risk of type 2 diabetes in individuals with obesity [35,36]. Isoleucine was positively correlated with *Eubacterium hallii* and *Fusicatenibacter*, consistent with previous studies [11,37]. In particular, *Eubacterium* has been associated with high protein intake, which correlates with increased BCAA levels [38]. Therefore, these findings suggest that the gut microbiota promotes obesity associated with circulating BCAA.

Inosine and uric acid, which are products of purine metabolism, were increased in participants with obesity. In mice that were fed a high-fat diet, these increased metabolites were associated with gut dysbiosis [39], decreasing uric acid degradation and thereby increasing its serum levels [40]. Excessive serum uric acid affects the metabolic system, causing fat accumulation in HepG2 cells and inducing mitochondrial oxidative stress: a key pathogenic mechanism of obesity [41,42].

We found that serum bile acids, especially TDCA and TMCA (α + β), were decreased in participants with obesity, consistent with previously reported studies, in which systemic TDCA levels decreased significantly in diet-induced obese animal models and high-fat diet subjects, and were restored after bariatric surgery [43,44]. An animal study revealed that TDCA was associated with obesity as an agonist of the Farnesoid X nuclear receptor, which regulates lipid, glucose, and energy metabolism [45]. Increased *Firmicutes* and decreased *Bacteroidetes* in adults with obesity have been indirectly associated with bile acid resistance [46]. Our results suggest that gut microbial alterations associated with bile acid profiles contribute to obesity and metabolic disturbances.

We performed an obesity intervention program to identify metabolic pathways with therapeutic relevance. Interestingly, fecal myristic acid increased significantly in participants with obesity at baseline. However, urinary myristic acid decreased significantly after the lifestyle intervention. A significant correlation was observed between urinary myristic acid and *Bacteroides* in the responder group. Long-chain fatty acids, such as myristic acid, affect microbial composition by inhibiting bacterial growth, which in turn is associated with microbial dysbiosis [47]. Consistently, another study revealed that in mice, the chronic administration of dietary myristic acid aggravated obesity-associated insulin resistance with a hypercholesterolemic effect [48]. Our findings also indicate a key role for myristic acid in de novo fatty acid biosynthesis, which may affect weight loss in pathway analysis. We found that ALT levels increased in participants with obesity. Since long-chain fatty acids, including myristic acid, stimulate the excessive influx of fatty acids into hepatocytes and induce the increase of pro-inflammatory cytokines and NF-κB, contributing to hepatic inflammation, our findings identified a previously unknown link between microbial and metabolomic alterations in obesity, suggesting myristic acid as a possible target for treating pediatric obesity.

Overall, the levels of intermediates in fatty acid biosynthesis were significantly lower in patients who responded to the intervention. Increased fatty acid biosynthesis positively correlates with BMI [49]. In the murine *ob*/*ob* model, genes related to fatty acid biosynthesis were upregulated, so increased fatty acid biosynthesis likely contributed to increased TG [50]. Additionally, this metabolic pathway is relevant to gut microbial function [51]. Functional microbiota pathways in obese rodents included an enriched fatty acid biosynthesis [9,51]. Thus, decreased fatty acid biosynthesis may have resulted in increased weight loss in the responder group. In metabolite profiles corresponding to inferred functional metabolic analysis of gut microbiota in a previous study [21], decreased methionine led to weight loss by increasing energy expenditure and improving glucose tolerance [52].

Several integrative interventional studies on obesity have combined metabolites and microbiota; however, most were conducted in adults and not children. To our knowledge, this is the first integrative interventional study to investigate alterations in the gut microbiota and metabolites in the serum, urine, and feces of participants with pediatric obesity. Our findings suggest a possible pathway for weight reduction and therapeutic targets.

Our study has several limitations. We identified significantly altered metabolites after the intervention; however, their numbers were small. These findings may be a consequence of the small number of participants, a short period of intervention, and a controlled before-and-after study design. A longitudinal randomized controlled study with larger sample sizes is required in the future. Additionally, targeted metabolomics could be considered to validate the altered metabolites associated with obesity in this study. Because the 16S rRNA gene amplicon sequencing for metagenome analysis identifies genus-level changes, species-level changes and actual functional profiling could not be identified. Finally, this study was limited to Korean children, and our findings should be validated in other ethnicities and adults. To further confirm the role of gut microbiota in obesity, additional in vitro experimental studies are required to demonstrate the production and transport of these altered metabolites.

## 5. Conclusions

This study investigated the metabolic pathways associated with pediatric obesity, and the effects of lifestyle interventions using metabolomic and metagenomic approaches. The most distinct metabolic alterations in the obese group were BCAA and purine changes. The lifestyle interventions resulted in obesity-related microbial and metabolic alterations in the responder group, including a decreased abundance of *Bacteroides* and urinary myristic acid concentrations, which suggest weight loss occurred by reduced fatty acid biosynthesis. These results could be valuable for identifying novel targets and biomarkers for the treatment of obesity.

## Figures and Tables

**Table 1:** Anthropometric measurements and biochemical blood markers of the study participants (*n* = 58).

	Normal Weight (*n* = 22) ^1^	Responder (*n* = 17)		Non-Responder (*n* = 19)	
		Pre	Post	Pre	Post
Male/Female	14/8	10/7		11/8	
Age (years)	10.24 ± 2.23	10.05 ± 2.29		10.34 ± 2.59	
**Anthropometric measurements**					
Weight (kg)	34.57 ± 8.68 ^3^	57.5 ± 16.39	57.38 ± 16.22	61.03 ± 21.3	62.53 ± 21.49 ^5^
Weight (z-score)	−0.48 ± 1.09 ^3^	2.38 ± 0.74	2.29 ± 0.75 ^5^	2.46 ± 0.97	2.49 ± 0.99
Height (cm)	143.7±12.19	145.76 ± 14.35	146.78 ± 14.16 ^5^	148.73 ± 14.23	149.75 ± 14.2 ^5^
Height (z-score)	0.09 ± 0.87 ^2^	0.99 ± 0.99	1.00 ± 1.00	1.29 ± 0.95	1.29 ± 0.86
BMI (kg/m^2^)	16.82 ± 2.15 ^3^	26.41 ± 3.92	26.01 ± 3.88 ^5^	26.64 ± 5.03	26.94 ± 5.14 ^4^
BMI (z-score)	−0.75 ± 1.05 ^3^	2.65 ± 0.79	2.50 ± 0.80 ^5^	2.60 ± 1.03	5.36 ± 11.54
Systolic BP (mmHg)	98.05 ± 9.19 ^3^	119.71 ± 13.33	118.18 ± 9.06	121.05 ± 11.39	119.26 ± 11.64
Diastolic BP (mmHg)	60.38 ± 5.32 ^3^	75.88 ± 7.81	75.29 ± 9.94	72.05 ± 10.04	73.63 ± 10.44
Waist circumference (cm)	58.94 ± 4.31 ^3^	86.14 ± 11.02	84.31 ± 10.45 ^5^	88.81 ± 12.9	90.07 ± 13.28 ^4^
Waist-to-height ratio	0.45 ± 0.03 ^3^	0.59 ± 0.05	0.57 ± 0.04 ^5^	0.60 ± 0.05	0.60 ± 0.05
Total body fat (%)	20.69 ± 8.63 ^3^	39.63 ± 4.99	38.35 ± 4.8 ^5^	38.79 ± 5.03	39.34 ± 4.93
Skeletal muscle mass (kg)	10.12 ± 4.11 ^3^	18.38 ± 5.96	24.16 ± 17	19.84 ± 7.63	20.25 ± 7.66 ^4^
**Blood biochemical profiles**					
Glucose (mg/dL)	95.67 ± 5.51 ^2^	100.53 ± 11.05	102.18 ± 9.28	106.74 ± 18.17	100.32 ± 7.4
AST (IU/L)	28.24 ± 5.14	26.65 ± 9.72	24.12 ± 7.21	26.26 ± 8.66	27.42 ± 12.49
ALT (IU/L)	15.76 ± 6.45 ^3^	30.82 ± 24.51	25.94 ± 19.12 ^4^	32.16 ± 21.46	33.95 ± 26.75
Triglyceride (mg/dL)	60.76 ± 27.78 ^3^	110.82 ± 64.9	101.12 ± 57.48	95.47 ± 38.72	100.63 ± 45.09
HDL cholesterol (mg/dL)	61.52 ± 12.06 ^3^	50.94 ± 10.84	51.18 ± 10.35	52.47 ± 9.84	53.37 ± 10.58
LDL cholesterol (mg/dL)	92.24 ± 18.03 ^2^	108.29 ± 23.29	107.06 ± 26.23	102.32 ± 21.59	104.74 ± 18.82
hs-CRP (mg/L)	0.46 ± 0.53 ^3^	2.45 ± 2.83	1.88 ± 1.91	1.59 ± 1.56	2.02 ± 2.26

Data are expressed as means ± standard deviation (SD). BMI, body mass index; AST, aspartate aminotransferase; ALT, alanine aminotransferase; HDL cholesterol, high-density lipoprotein cholesterol; LDL cholesterol, low-density lipoprotein cholesterol; hs-CRP, high sensitivity C reactive protein; Pre, pre-intervention; Post, post-intervention. ^1^ In 22 normal-weight participants, 21 serum samples were collected. ^2^ *p* < 0.05 and ^3^ *p* < 0.01; normal group vs. obese group. ^4^ *p* < 0.05 and ^5^ *p* < 0.01; pre vs. post in obese group.

## Data Availability

All raw 16S rRNA gene sequencing data have been deposited in the NCBI Sequence Read Archive under accession number SUB7459302 (BioProject PRJNA633584). Metabolomics data are available in the electronic Supplementary Files and at the NIH Common Fund’s National Metabolomics Data Repository website (Project ID: PR001443). These data will be made available upon publication.

## Supplementary Materials

The following supporting information can be downloaded at: https://www.mdpi.com/article/10.3390/nu15092184/s1, Figure S1: Principal component analysis (PCA) between normal-weight and obese groups.; Table S1: List of metabolites and fold change in the obese group compared to that in the normal group.; Table S2: List of metabolites and changes in their content after lifestyle intervention; Table S3: Metabolite involved in the functional metabolic pathway of the gut microbiota.
