# Supplementary material for: Serum, Urine, and Fecal Metabolome Alterations in the Gut Microbiota in Response to Lifestyle Interventions in Pediatric Obesity: A Non-Randomized Clinical Trial
Source: Nutrients. 2023 May 4;15(9):2184. doi: 10.3390/nu15092184 (PMC10180968; doi:10.3390/nu15092184)
Supplement: Supplementary file 1 [file nutrients-15-02184-s001.zip › nutrients-2369560-supplementary.pdf]

**Serum, urine, and fecal metabolome alterations in the gut microbiota in response to lifestyle interventions in pediatric obesity**

## Supplementary Materials

**Table S1**. List of metabolites and fold change in the obese group compared to that in the normal group.

**Table S2**. List of metabolites and changes in their content after lifestyle intervention.

**Table S3**. Metabolites involved in the functional metabolic pathway of the gut microbiota.

**Figure S1.** Principal component analysis (PCA) between normal-weight and obese groups. Score plots of the PCA model for (**a**) serum, (**b**) urine, and (**c**) feces. N, normal; Ob, obese.

# Serum, urine, and fecal metabolome alterations in the gut microbiota in response to lifestyle interventions in pediatric obesity

**Table S1.** Metabolites altered in obesity.

| Name | Fold Change | *P*-value | FDR-adjusted *P*-value | Class |
|---|---|---|---|---|
| **Serum marker** | | | | |
| L-Isoleucine | 1.40 | $7.4 \times 10^{-9}$ | $5.6 \times 10^{-8}$ | Branched-chain amino acids |
| L-Lysine | 1.40 | $5.9 \times 10^{-10}$ | $5.7 \times 10^{-9}$ | essential amino acid |
| Pimelic acid | 0.52 | $1.1 \times 10^{-10}$ | $1.2 \times 10^{-9}$ | Fatty acyls |
| Suberic acid | 0.39 | $2.2 \times 10^{-10}$ | $2.3 \times 10^{-9}$ | Fatty acyls |
| Uric acid | 1.52 | $2.5 \times 10^{-6}$ | $1.1 \times 10^{-5}$ | Imidazopyrimidines |
| Inosine | 3.44 | $8.0 \times 10^{-9}$ | $6.0 \times 10^{-8}$ | Purine nucleosides |
| TDCA | 0.28 | $7.5 \times 10^{-5}$ | $7.5 \times 10^{-5}$ | Bile acid |

| | | | | |
|---|---|---|---|---|
| TMCA (α + β) | 0.17 | $2.2 \times 10^{-6}$ | $2.5 \times 10^{-6}$ | Bile acid |
| **Urinary marker** | | | | |
| L-Cystine | 1.60 | $5.1 \times 10^{-4}$ | $4.0 \times 10^{-3}$ | Amino acids |
| 2,4-Dihydroxybutanoic acid | 0.68 | $2.5 \times 10^{-4}$ | $2.3 \times 10^{-3}$ | Fatty acids and conjugates |
| Ribonic acid | 0.52 | $3.5 \times 10^{-4}$ | $3.0 \times 10^{-3}$ | Fatty acids and conjugates |
| 2,3-Dihydroxybutanoic acid | 2.03 | $9.4 \times 10^{-8}$ | $8.6 \times 10^{-6}$ | Carbohydrate conjugates |
| Adonitol | 1.50 | $6.8 \times 10^{-3}$ | $2.9 \times 10^{-2}$ | Carbohydrate conjugates |
| Glyceric acid | 0.54 | $2.5 \times 10^{-4}$ | $2.3 \times 10^{-3}$ | Carbohydrate conjugates |
| Threonic acid | 0.62 | $9.2 \times 10^{-4}$ | $6.3 \times 10^{-3}$ | carbohydrate conjugates |
| **Fecal marker** | | | | |
| Myristic acid | 2.21 | $1.4 \times 10^{-2}$ | $3.0 \times 10^{-1}$ | Long-chain fatty acids |

| | | | | |
|---|---|---|---|---|
| Putrescine | 0.27 | $5.0 \times 10^{-2}$ | $4.4 \times 10^{-1}$ | Amines |

TDCA, Taurodeoxycholic acid; TMCA (α + β), Tauromuricholic acid (alpha and beta); FDR, False discovery rate.

**Serum, urine, and fecal metabolome alterations in the gut microbiota in response to lifestyle interventions in children with obesity**

**Table S2.** Altered metabolites after lifestyle intervention.

| Group | Name | Fold Change | *P*-value | FDR-adjusted *P*-value | Class |
|---|---|---|---|---|---|
| **Serum marker** | | | | | |
| R pre vs. post | Oxalic acid | 1.16 | $2.2 \times 10^{-2}$ | $2.0 \times 10^{-1}$ | Carboxylic acids and derivatives |
| | Nonanoic acid | 0.88 | $4.7 \times 10^{-3}$ | $1.3 \times 10^{-1}$ | Fatty acyls |
| | Arachidic acid | 1.08 | $1.1 \times 10^{-2}$ | $1.7 \times 10^{-1}$ | Fatty acyls |
| | 2-Hydroxybutyric acid | 0.85 | $3.6 \times 10^{-2}$ | $2.2 \times 10^{-1}$ | Hydroxy acids and derivatives |
| | 3,4-Dihydroxybutyric acid | 1.11 | $4.8 \times 10^{-2}$ | $2.2 \times 10^{-1}$ | Hydroxy acids and derivatives |
| **NR pre vs. post** | **No markers** | | | | |
| **Urinary marker** | | | | | |

| | | | | | |
|---|---|---|---|---|---|
| **R pre vs. post** | Ribonic acid | 1.38 | $4.2 \times 10^{-2}$ | $7.0 \times 10^{-1}$ | Carbohydrates |
| | Myristic acid | 0.69 | $7.7 \times 10^{-6}$ | $1.2 \times 10^{-3}$ | Long-chain fatty acid |
| **NR pre vs. post** | Hippuric acid | 0.50 | $1.5 \times 10^{-2}$ | $1.2 \times 10^{-1}$ | Benzoic acids and derivatives |
| | Inosine | 0.77 | $2.6 \times 10^{-2}$ | $1.6 \times 10^{-1}$ | Purine nucleosides |
| **Fecal marker** | | | | | |
| **R pre vs. post** | Picolinic acid | 0.74 | $3.1 \times 10^{-2}$ | $7.8 \times 10^{-1}$ | Pyridines and derivatives |
| **NR pre vs. post** | | | **No markers** | | |

R, responder; NR, non-responder; pre, pre-intervention; post, post-intervention; FDR, False discovery rate.

Direction of comparison for fold change: post-intervention/pre-intervention.

**Table S3.** Metabolite involved in functional metabolic pathway of gut microbiota.

| Name | Fold Change | *P*-value | FDR adjusted *P*-value | Sample type | Functional metabolic pathway |
|---|---|---|---|---|---|
| Methionine | 0.91 | $4.1 \times 10^{-2}$ | $2.2 \times 10^{-1}$ | Serum | Aspartate superpathway |

FDR, False discovery rate.

**Serum, urine, and fecal metabolome alterations in the gut microbiota in response to lifestyle interventions in children with obesity**

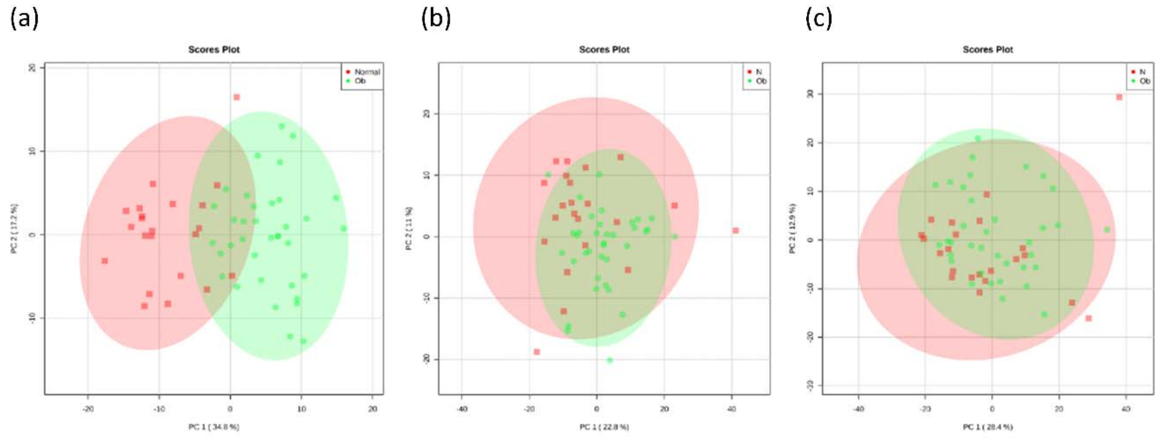


**Figure S1.** Principal component analysis (PCA) between normal-weight and obese groups. Score plots of the PCA model for (**a**) serum, (**b**) urine, and (**c**) feces. N, normal; Ob, obese.
